# Arecoline promotes proliferation and migration of human HepG2 cells through activation of the PI3K/AKT/mTOR pathway

**DOI:** 10.1186/s41065-022-00241-0

**Published:** 2022-07-14

**Authors:** Hai Xie, Ren Jing, Xiaoting Liao, Haishao Chen, Xianlong Xie, Huijun Dai, Linghui Pan

**Affiliations:** 1 Department of Anesthesiology, Guangxi Medical University Affiliated Cancer Hospital, He Di Rd No.71, Nanning, 530021 PR China; 2 Guangxi Key Laboratory for Basic Science and Prevention of Perioperative Organ Disfunction, Guangxi Medical University Cancer Hospital, Nanning, China; 3 Department of Anesthesiology, The First Affiliated Hospital of Hainan Medical College, Haikou, China

**Keywords:** Arecoline, Hepatocellular cancer, Cell proliferation, Cell migration, PI3K/AKT/mTOR pathway, CDK1

## Abstract

**Background:**

Arecoline is a well-known risk factor for oral submucosal fibrosis and cancer. However, the mechanistic correlation between arecoline and hepatocellular cancer remains elusive. Here, we investigated the effect of arecoline on the proliferation and migration of human HepG2 hepatoma cells and its potential oncogenic mechanisms.

**Methods:**

Bioinformatic technologies were used to identify the deferentially expressed miRNAs (DE-miRNAs) and hub target genes of arecoline-induced cancers. These DE-miRNAs, hub genes and pathway were proved in arecoline-treated HepG2 cells.

**Results:**

A total of 86 DE-miRNAs and 460 target genes were identified. These target genes are associated with DNA-templated regulation of transcription and other biological processes. Significant molecular functions were protein binding, calcium ion binding, and enrichment in the nucleus and cytoplasm. These genes are involved in the PI3K-AKT pathway. CDK1, CCND1, RAF1, CDKN1B and BTRC were defined as the top 5 hub target genes, and patients with high expression of CDK1 showed poor prognosis. Compared with control group, 2.5 µM arecoline treatment increased the proliferation and migration ability of the HepG2 cells. Treatment with 2.5 µM arecoline increased the levels of miR-21-3p, miR-21-5p and miR-1267, upregulated the expression of PI3K-AKT pathway factors, CDK1, CCND1 but decreased RAF1 expression.

**Conclusion:**

A low concentration arecoline can induce the proliferation and migration of HepG2 cells, with the potential mechanism of action linked to high levels of exosomal miR-21 and miR-1267, activation of the PI3K-AKT pathway, upregulation of CDK1 and CCND1, and downregulation of RAF1.

## Introduction

The areca nut is rich in alkaloids such as arecoline, a widely used stimulant akin to tobacco, alcohol, and caffeine [1]. Although arecoline has some medical benefits in the removal of parasites, treatment of bacterial infection and prevention of influenza, its cytotoxic qualities can cause apoptosis of human oral epithelial cells, endothelial cells and umbilical vein endothelial cells, thereby promoting oral submucous fibrosis [2, 3]. Long-term chewing of betel quid can cause varying degrees of damage to the oral mucosa, buccal epithelial, immune cells and reproductive function, and can even lead to addiction [4–6].

Arecoline is a known risk factor for oral cancer and a potential risk factor for cirrhosis and hepatocellular cancer (HCC). Treating mice with arecoline leads to decreased nuclear size, inflated cysternae of the endoplasmic reticulum, abundant lipid droplets, and an increased in serum levels of the hepatotoxic marker enzymes glutamic-oxaloacetic transaminase and glutamic-pyruvic transaminase [7]. Arecoline can also induce liver-damaging activity of cytochrome p450 2E1 (CYP2E1) activity to damage liver, suggesting that patients with areca nut hobby may have risk of metabolic interaction when they received drug therapy using CYP2E1 as substrate [8], and it can activate p53 and its downstream p21WAF1 to damage DNA and induce apoptosis [9, 10]. Arecoline can induce anoikis in HA22T/VGH cells by inhibiting of STAT3 and activating RhoA/Rock [11]. Despite these studies, much remains to be clarified about how arecoline may contribute to HCC.

MicroRNAs (miRNAs) are non-coding small RNAs that regulate gene expression and that are frequently dysregulated in tumorigenesis. They may therefore serve as potential cancer biomarkers for cancer diagnosis and prognosis [12–14]. In the present study, we used bioinformatics approaches to analyze the miRNA expression data of arecoline-induced cancer from the Gene Expression Omnibus (GEO) database, and identified the target genes of the differentially expressed miRNAs (DE-miRNAs) compared to healthy volunteers. We performed Kyoto Encyclopedia of Genes and Genomes (KEGG) and hub target genes analysis to illuminate potential biomarkers and related pathways in arecoline-induced cancer. We further investigated the roles of these predicted markers by treating human hepatoma HepG2 cultures with arecoline.

## Materials and methods

### Microarray dataset

We compared the genome-wide miRNA expression profiles of whole blood-derived RNA samples between patients with arecoline-induced cancer and healthy volunteers using the raw data in the NCBI GEO databases (http://www.ncbi.nlm.nih.gov/geo). The microarray expression dataset GSE45238 had been obtained with the Affymetrix GPL8179 platform (Illumina Human v2 MicroRNA expression bead chip), which was submitted by Shiah SG et al. [15, 16] between March 18, 2013 and October 6, 2020. Here we focused on the DE-miRNAs related to cell proliferation, migration and invasion.

### Identification of DE-miRNAs and their target genes

The miRNA expression data were read and normalized using R software (version: × 64 3.2.1) and the “Affy”, “Limma” and “Impute” packages. Missing values were treated via the k-nearest neighbor method. The normalized data were used to screen for DE-miRNAs between the patients and healthy volunteers, with a criterion of fold change (FC) > 2 and adjusted *p* < 0.05. Heat and volcano maps of DE-miRNAs were constructed using pheatmap and Ggplot2 packages. The target genes of DE-miRNAs were predicted using Targetscan (http://www.targetscan.org/vert_72/), with inclusion criteria of cumulative weighted context +  + score < -0.3, and aggregate P_CT_ > 0.80). For target genes without an aggregate P_CT_, the inclusion criterion was cumulative weighted context +  + score < -0.6.

### Functional annotation of target genes using GO and KEGG method

The GO database provides a framework for the characterization of biological data from high-throughput genomes studies [17]. The expression matrix of all target genes was uploaded to the DAVID (version 6.8; https://david.ncifcrf.gov/) database to obtain the GO terms, including biological processes (BPs), cellular components (CCs) and molecular functions (MFs). KEGG pathway analysis provides cell pathways enrichment functional information [18]. All target genes were submitted to DAVID to perform the GO enrichment and KEGG pathway analysis with count > 5 and *p* < 0.01. Enrichment results were visualized by the enrichment dot bubbles and GO chord using R software.

### Analysis of protein–protein interactions (PPI) and hub-target genes

The STRING database was used to assess the interactive relationships of all identified target genes, and the experimentally validated interactions were defined as statistically different when the combined score ≥ 0.4. The hub target genes, defined as hub genes with degree ≥ 10, were then identified using the cyto-Hubba application in Cytoscape software (version 3.6.1). Subsequently, functional analysis including GO enrichment and KEGG pathway analysis of the hub genes was performed using the DAVID database. Functional information about hub genes was also obtained from the UniProt database (https://www.UniProt.org/). The predicted expression of the top 5 hub genes in HCC was analyzed via Gene Expression Profiling Interactive Analysis.

### Cell Culture

The human hepatoma cancer cell line HepG2 was purchased from the Shanghai Institute of Biochemistry and Cell Biology, Chinese Academy of Sciences (Shanghai, China). HepG2 cells were cultured in RPMI-1640 medium (Sigma-Aldrich, Cat. R8758-500ML, St. Louis, MO, USA) containing 10% fetal bovine serum (Gibco, Cat. A3160802, Carlsbad, CA, USA), 1.5 g/L NaHCO_3_, 20 mM L-glutamine and 1% penicillin–streptomycin (Gibco, Cat. 15,070,063, Carlsbad, CA, USA) at 37℃ in a humidified atmosphere with 5%CO_2_.

### Cell viability assay

Cells were seeded in a 96-well plate and incubated for 24 h, followed by treatment with 0–10 μM arecoline (Sigma-Aldrich, St. Louis, MO, USA) for 1, 2, 3, 5 and 7 days. The Cell Counting Kit (CCK)-8 kit (Elabscience Biotechnology Cat. E-CK-A362, Wuhan, China) was used according to the manufacturer’s instructions to quantify cell proliferation. Based on the results of CCK8 tests in pilot studies (data not shown), we decided on a final arecoline concentration for further experiments.

### Cell ultrastructure and inflammation

Following arecoline treatment, cell ultrastructure was observed by transmission electron microscopy. Levels of the inflammatory markers interleukin (IL)-1β (CUSABIO, Cat. CSB-E08053h, Wuhan, China) and tumor necrosis factor (TNF)-α (CUSABIO, Cat. CSB-E04740h, Wuhan, China) in cell culture medium were assessed by enzyme-linked immunosorbent assay.

### Cell apoptosis and cycle assay

Cells apoptosis and cycle were assessed using the Annexin V-AF647/PI kit (Invitrogen, Cat. 331,200, Waltham, MA, USA) and cell cycle assay kit (Invitrogen, Cat. A10798), respectively, according to the manufacturer’s instructions. Briefly, the arecoline-treated cells were collected, washed with pre-chilled phosphate buffered saline (PBS), suspended in binding buffer at a density of 2 × 10^6^ cells/mL, then stained with 5 µL of Annexin-V FITC and 10 µL propidium iodide (PI). The cell suspension was incubated in the dark at room temperature for 15 min. To determine the distribution of cells in each phase of the cell cycle, the arecoline-treated cells were suspended in 1 mL binding buffer at a density of 1 × 10^6^ cells/mL, fixed with 500 μL chilled ethanol (70%) for 2 h to overnight, then washed again with PBS. The cells were incubated in 500 μL solution of PI and RNase A (1:9), followed by incubation for 45 min in the dark at room temperature. Cell apoptosis and cell cycle distribution were analyzed using a BD FACS Calibur™ flow cytometer (BD Biosciences, NJ, USA).

### Cell migration assay

A wound healing experiment was used to evaluate the cell migration capacity of arecoline-treated HepG2 cells. Briefly, 5 × 10^5^ cells/well were cultured in 6-well plates and incubated for 24 h at 37 °C. A wound was created with a scratch using a sterile 10 μL pipette tip after the cells reached confluence. The cells were then washed with PBS to clear the detached cells and evaluated at 1 d, 3 d and 7 days later. The invasive properties of the arecoline-treated HepG2 cells were determined using a Transwell experiment. The matrigel gel (Corning, Cat. 356,234, NY, USA) and serum-free RIpM-1640 medium were mixed in a 3:1 ratio, then adding 60 μL mixture to each Falcon® permeable Support for 6-well plate with 0.4 µm Transparent PET Membrane (Corning, Cat. 353,090). Cells (1 × 10^6^) in 100 μL medium were seeded into the upper chamber and then 600 μL RPMI-1640 medium with 10% FBS was added for a 1-day incubation. The cells able to migrate through the pores to the other side of the membrane and were stained and counted.

### Exosomes isolation

Exosomes were isolated from cell culture medium using the “Total Exosome Isolation kit (from cell culture media)” (Invitrogen, Cat. 4,478,359) according to the manufacturer’s instructions. Briefly, the culture medium was centrifuged at 2000 × g for 30 min to remove cells and debris. The cell-free supernatant was then transferred to a new tube and treated with 0.5 volumes of the total exosome isolation reagent. After adequate mixture by vortexing, the samples were incubated at 2–8 ℃ overnight. The samples were then centrifuged at 10, 000 × g for 1 h at 2–8 ℃, and the exosome-containing pellets were resuspended in a convenient volume of PBS.

### Total RNA isolation

Total RNA was isolated from exosomes using the Total Exosome RNA and Protein Isolation Kit (Invitrogen, Cat. 4,478,545) according to the manufacturer’s instructions. Briefly, a total of 200 μL exosome sample was resuspended by 1X PBS at an RNase-free tube, and added one volume of 2X Denaturing Solution, followed by incubation for 5 min on ice. The samples were then treated with one volume of Acid-Phenol: Chloroform, mixed by vortexing for 30–60 s and centrifuged at 12, 000 × g for 5 min at room temperature. The supernatant in the upper layer (about 300 μL) was transferred to a new tube. To purify the total RNA including miRNA, 1.6 volumes of 100% ethanol was added, and the mixture was centrifuged at 4,000 × g for 1 min at 4 ℃. Wash buffers 1 and 2 (500 μL each) were sequentially used to wash the RNA, and the mixtures were centrifuged at 12, 000 × g for 1 min at 4 ℃. An appropriate volume of elution buffer was finally used to elute the RNA from the column after an incubation of 2–5 min at room temperature. The concentration and purification of RNA were assessed using a standard laboratory spectrophotometer.

### Real time-quantitative PCR of miR-21-3p, miR-21-5p and miR-1267 expression

Reverse transcription and PCR were performed with the All-in-One™ miRNA RT-qPCR Detection Kit 2.0 (GeneCopoeia, Cat. QP115, Guangzhou, China) according to the manufacturer’s instructions. Briefly, the first-strand cDNA was synthesized 5 ng to 1 μg of total RNA or 0.1 ng to 1 μg of miRNA, 1 μL of 2 U/μL poly A Polymerase, 1 μL of 20 × SureScriptTM RTase Mix, 4 μL of 5 × PAP/RT Buffer II, and ddH_2_O up to 20 μL. The mixture was incubated for 60 min at 37 ℃ and 5 min at 85 ℃.

PCR was carried out using 10 μL of 2 × All-in-One™ qPCR Mix, 2 μL of 2 μM All-in-One™ miRNA qPCR primer, 2 μL of 2 μM Universal Adaptor PCR primer, 2 μL of first-strand cNDA (diluted 1:5), 0.2 μL of 30 μΜ ROX Reference Dye, and ddH_2_O up to 20 μL. Reaction conditions included pre-denaturation at 95 ℃ for 10 min (1 cycle), denaturation at 95 ℃ for 10 s, annealing at (Tm-2) ℃ for 20 s, and extension at 72 ℃ for 10 s (40 cycles). Relative miRNA expression was calculated using the 2^−ΔΔCt^ method against housekeeping miRNA (U6) expression as the internal reference.

### Real time-quantitative PCR for mRNA expression

Total RNA was isolated from arecoline-treated HepG2 cells using the Trizol reagent (Invitrogen, Cat. 15,596,018) according to the standard protocol. RNA concentration and purity were examined by spectrophotometry, and cDNA was synthesized using the PrimeScript™ II 1st Strand cDNA Synthesis Kit (Takara, Beijing, China). The primer sequences for GAPDH, AKT, PI3K, mTOR, CDK1, CCND1, RAF1, CDKN1B and BTRC were listed in Table 1. RT-qPCR was carried out using the PrimeScript™ RT reagent Kit with gDNA Eraser (perfect Real Time; Takara, Cat. RR047A, Beijing, China) according to the manufacturer’s instructions. Relative gene expression was determined using the 2^−ΔΔCt^ method against the housekeeping gene GAPDH as the internal reference.

**Table 1:** Primer sequences used to detect target mRNAs

Gene	Primer sequence (5’ → 3’)
AKT	F: ACA CTC CAC TCA CTC ACA CCT CTC R: GCA CAG CCA CAC CTA CAG CAC
PI3K	F: CGG TTG TTA AGG AAG AGG CGA CTC R: AGT GAC TCA GGC TGG TGG ATG G
mTOR	F: TGG TGC TCA TTG CCT GTG CTT AG R: GGT GGC GTG AAG TGA GTC TGT G
CDK1	F: CAC AAA ACT ACA GGT CAA GTG G R: GAG AAA TTT CCC GAA TTG CAG T
CCND1	F: GTC CTA CTT CAA ATG TGT GCA G R: GGG ATG GTC TCC TTC ATC TTA G
CDKN1B	F: CTA ACT CTG AGG ACA CGC ATT T R: TTG AGT AGA ATC GTC GGT T
RAF1	F: AA GAC AAG CAA CAC TAT CCG T R: CAG TAT TCC AAT CTA AGC GTG C
BTRC	F: CTG GAT GCC AAA TCA CTA TGT G R: GAT AAG CTT CCA CAG CAT G
GAPDH	F: CAG GAG GCA TTG CTG ATG AT R: GAA GGC TGG GGC TCA TTT

### Western blotting analysis

Total proteins were extracted from arecoline-treated HepG2 cells, and the concentration was determined by a standard BCA assay (Thermo Fisher Scientific, Cat. A53227, Waltham, MA, USA). Molecular weight marker (Thermo Fisher Scientific, Cat. 26,616) and samples were fractionated by SDS–Polyacrylamide gel for electrophoresis, then transferred onto a nitrocellulose membrane, which was blocked with 3% bovine serum albumin and Tris-buffered saline containing 0.1% Tween-20. Next, the blocked membranes were incubated at 4℃ overnight with primary antibodies (Table 2) against potential hub-target genes and GAPDH as an internal reference. Washed membranes were incubated with horseradish peroxidase-conjugated goat anti-rabbit secondary antibody (Abcam, cat. Ab6721, Cambridge, UK). protein bands were visualized using a Novex™ ECL Chemiluminescent Substrates (Invitrogen, Cat. WT20005) and semi-quantitatified using Image Lab 4.1 software (Bio-Rad, Hercules, CA, USA). Each protein’s expression was standardized with respect to an internal reference GAPDH.

**Table 2:** Antibodies used for Western blotting

Antibody	Manufacturer	Catalog no	Dilution
PI3 Kinase p85 (19H8) Rabbit mAb	CST	4257 T	1:1000
Phospho-PI3 Kinase p85 (Tyr458)/p55 (Tyr199) (E3U1H) Rabbit mAb	CST	17366S	1:1000
AKT (pan) (C67E7) Rabbit mAb	CST	4691 T	1:1000
Phospho-Akt (Ser473) (D9E) XP® Rabbit mAb	CST	4060S	1:2000
mTOR (7C10) Rabbit mAb	CST	2983 T	1:1000
Phospho-mTOR (Ser2448) Antibody	CST	2971S	1:1000
GAPDH (D16H11) XP® Rabbit mAb	CST	5174S	1: 1000
Anti-CDK1 (phospho T161) mAb	Abcam	ab201008-10 μl	1:1000
Anti-Cyclin D1 (CCND1) antibody	Abcam	ab40754-10 μl	1:1000
Anti-Raf1 antibody	Abcam	ab181115	1:1000
Anti-p27 KIP 1 (CDKN1B) antibody	Abcam	ab32034	1:1000
Anti-BTRC polyclonal Antibody	Solarbio	K002903p	1:500

*CST* Cell Signaling Technology, *mAb* Monoclonal antibody

### Statistical analysis

Data were analyzed using SPSS 22.0 software. All quantitative data were reported as mean ± SD. Differences between control and experimental groups were assessed for significance using the independent-sample t test. Statistical difference was defined as *p < 0.05, **p < 0.01, and ***p < 0.001.

## Results

### Identification of DE-miRNAs and GO and KEGG analysis of target genes in patients with arecoline-induced cancer and healthy controls

**Fig. 1:**
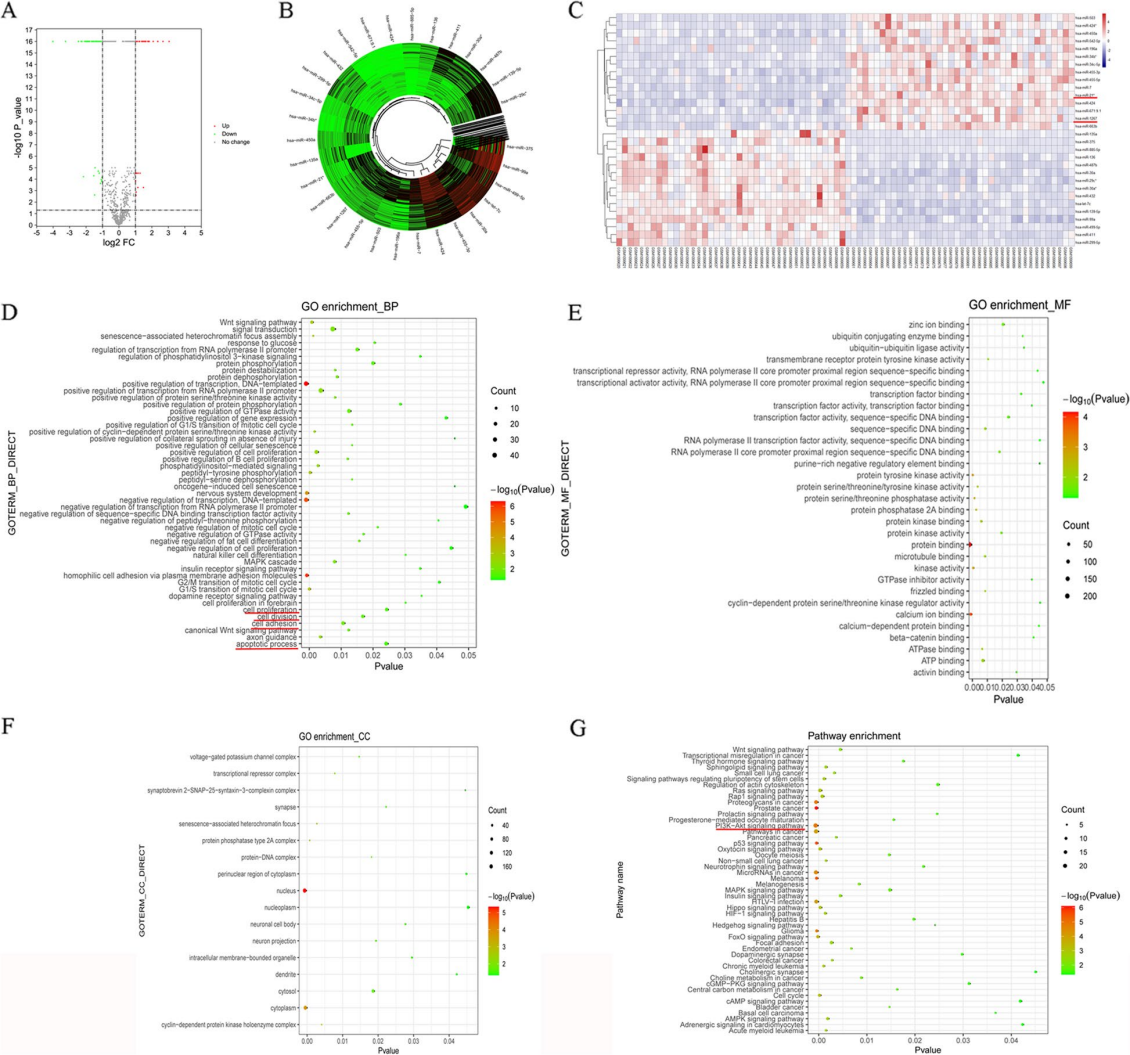
Expression, GO and KEGG analysis of DE-miRNAs in arecoline-induced cancer. **A** Volcanic plot of the GSE45238 data comparing patients with arecoline-induced cancer and healthy controls. Significantly upregulated miRNAs are indicated with red dotes, while downregulated miRNAs are indicated with green dotes. The dashed line illustrates the cut-off for significance (*p* < 0.01; T-test). **B** Circular cluster map. Upregulated miRNAs are indicated with red dotes, while downregulated miRNAs are indicated with green dotes. **C** Heat map of the top 15 up- or downregulated miRNAs. Red markers upregulation; blue, downregulation. **D** Enrichment of DE-miRNAs in GO biological processes (BPs). **E** Enrichment of DE-miRNAs in GO molecular functions (MFs). **F** Enrichment of DE-miRNAs in GO cellular components (CCs). **G** KEGG pathway enrichment analysis of DE-miRNAs. Node color and size reflect the log_10_ (*p* value) and count, respectively

To gain insights into the mechanisms by which arecoline may contribute to cancer, we screened the GSE45238 dataset for DE-miRNAs between patients and healthy volunteers. The results of this analysis are summarized in a volcano plot (Fig. 1A) and included 36 significantly upregulated and 50 downregulated DE-miRNAs. The circular cluster map and heat map of the top 15 upregulated and downregulated DE-miRNAs are displayed in Fig. 1B and C (Tables 3 and 4), respectively. A total of 563 potential DE-miRNAs target genes were identified through TargetScan, with 460 target genes deleted after duplication.

**Table 3:** Top 15 upregulated DE-miRNAs

miRNA	logFC	AveExpr	t	*P*	adj.P.Val	B	No. Of predicted target genes
hsa-miR-21	2.676	9.096	15.499	0.000	0.000	48.382	12
hsa-miR-503	2.365	9.700	13.265	0.000	0.000	39.467	58
hsa-miR-7	1.841	10.900	12.451	0.000	0.000	36.061	33
hsa-miR-455-3p	1.375	11.973	11.419	0.000	0.000	31.637	0
hsa-miR-1267	1.555	9.646	10.661	0.000	0.000	28.321	18
hsa-miR-455-5p	1.401	9.872	10.334	0.000	0.000	26.874	0
hsa-miR-424*	1.589	9.563	9.955	0.000	0.000	25.188	102
hsa-miR-34b	2.080	7.805	9.539	0.000	0.000	23.332	0
hsa-miR-663b	1.604	9.311	9.235	0.000	0.000	21.971	25
hsa-miR-450a	1.220	9.053	8.435	0.000	0.000	18.386	2
hsa-miR-34c-5p	1.573	8.506	8.406	0.000	0.000	18.256	102
hsa-miR-542-5p	1.785	7.023	8.385	0.000	0.000	18.159	26
hsa-miR-196a	3.044	8.903	8.057	0.000	0.000	16.695	32
hsa-miR-671:9.1	1.049	6.861	8.043	0.000	0.000	16.632	0

*adj. P* Adjust *p* value, *AveExpr* Average value of gene expression, *FC* Fold change. Notes: * hsa-mir-424 is duplicate among the top 15 upregulated DE-miRNAs

**Table 4:** Top 15 downregulated DE-miRNAs

miRNA	logFC	AveExpr	t	*P*	adj.P.Val	B	No. Of predicted target genes
hsa-miR-7c	-1.567	11.806	-13.761	0.000	0.000	41.502	4
hsa-miR-139-5p	-1.818	10.184	-11.795	0.000	0.000	33.262	0
hsa-miR-29c	-1.383	10.247	-11.437	0.000	0.000	31.715	0
hsa-miR-30a	-1.971	10.397	-10.981	0.000	0.000	29.231	32
hsa-miR-375	-4.013	9.975	-10.787	0.000	0.000	28.873	0
hsa-miR-411	-2.452	9.433	-10.325	0.000	0.000	26.837	0
hsa-miR-99a	-1.402	12.499	-9.755	0.000	0.000	24.297	0
hsa-miR-487b	-2.087	9.012	-9.626	0.000	0.000	23.722	1
hsa-miR-299-5p	-1.938	8.194	-9.477	0.000	0.000	23.054	2
hsa-miR-499-5p	-1.815	11.062	-9.300	0.000	0.000	22.262	0
hsa-miR-885-5p	-1.899	6.591	-9.227	0.000	0.000	21.936	6
hsa-miR-136	-1.903	7.836	-9.154	0.000	0.000	21.610	10
hsa-miR-135a	-2.249	7.677	-9.134	0.000	0.000	21.521	93
hsa-miR-432	-1.616	8.367	-8.888	0.000	0.000	20.414	17

*adj. p* Adjust *p* value, *AveExpr* Average value of gene expression, *FC* Fold change. Notes: Hsa-mir-30a is duplicated

GO analysis demonstrated that the identified target genes were significantly enriched in the BPs transcription regulation, DNA templated-templated regulation of transcription, homophilic cell adhesion, neural system development, positive regulation of cell proliferation and RNA polymerase II promoter transcription (Fig. 1D). The target genes were enriched in the MFs protein binding and calcium ion binding (Fig. 1E), as well as in the CCs nucleus and cytoplasm (Fig. 1F). KEGG pathway analysis showed that the predicted target genes were enriched in the PI3K-AKT pathway (Count = 23, *p* value < 0.001), which is the key signal pathway in arecoline-induced oncogenesis (Fig. 1G).

### PPI network construction and hub-target genes analysis

**Fig. 2:**
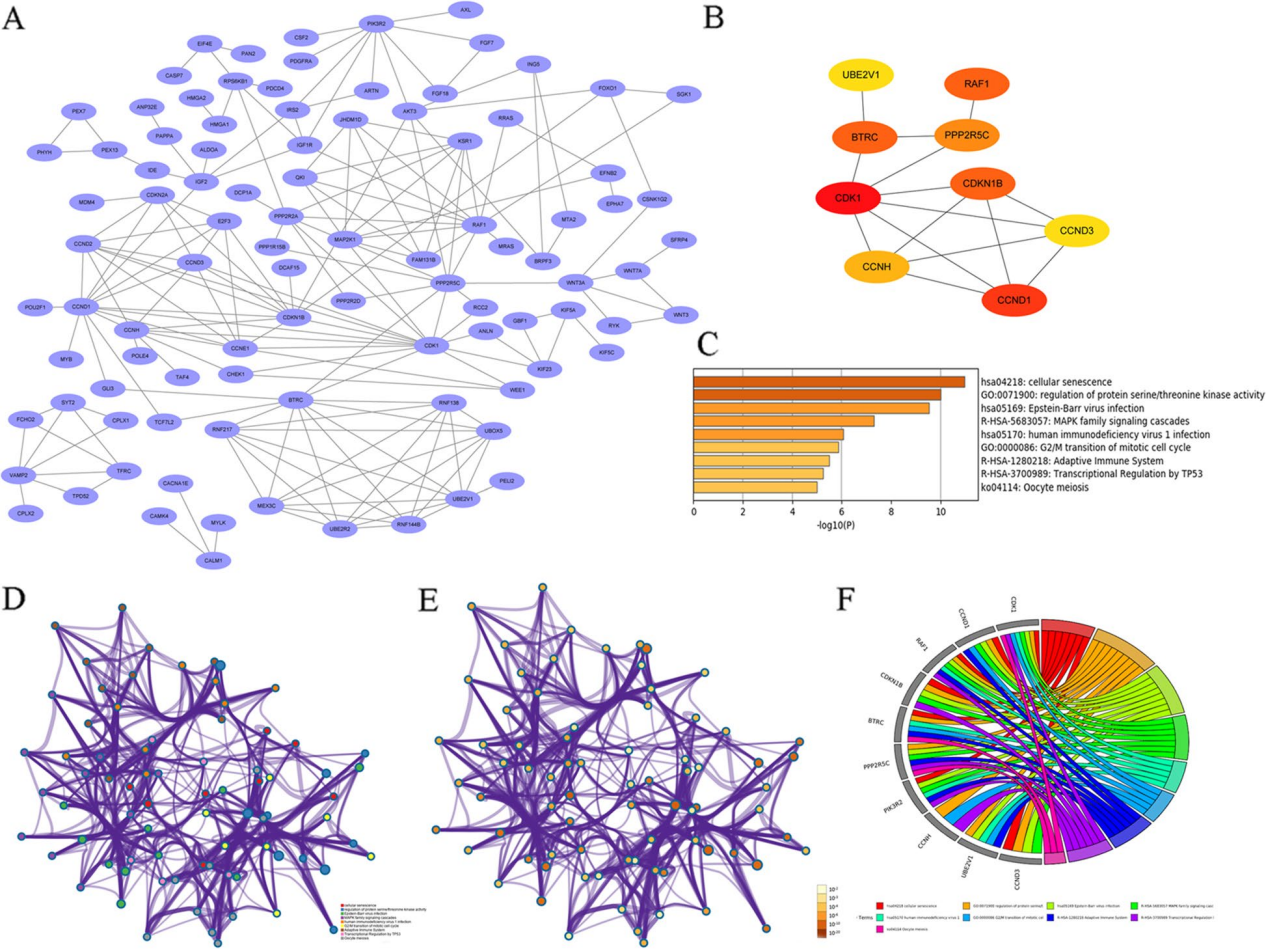
Protein–Protein interaction network and prediction of hub genes targeted by DE-miRNAs. **A** protein interaction network of all predicted DE-miRNA target genes. **B** protein interaction network of hub-target gene, with redder color indicating with higher degree of interaction. **C** Histogram of GO analysis of the identified hub-target genes. **D** Network of GO analysis classified by GO terms. **E** Network of GO analysis classified by *p* value. **F** GO chord of the identified hub-target genes

A PPI network of potential target genes to determine significant modules was constructed with 336 edges and 114 nodes (Fig. 2A). A total of 10 hub-target genes (hub genes) were identified, giving a PPI network with 14 edges and 10 nodes (Fig. 2B). GO analysis showed that these hub genes are involved in cellular senescence, regulation of protein serine/threonine kinase activity, Epstein-Barr virus infection, MAPK family signaling cascades, human immunodeficiency virus 1 infection, G2/M transition of mitotic cell cycle, among other terms (Fig. 2C-F). The hub genes were queried using the UniProt database (Table 5).

**Table 5:** Identified hub genes

Gene	Protein	UniProtKB ID	Biological process
CDK1	Cyclin-dependent kinase 1	p06493 (CDK1_HUMAN)	Apoptosis, Biological rhythms, Cell cycle, Cell division, Host-virus interaction, Mitosis
CCND1	G1/S-specific cyclin-D1	p24385 (CCND1_HUMAN)	Cell cycle, Cell division, DNA damage, Transcription, Transcription regulation
RAF1	RAF proto-oncogene serine/threonine-protein kinase	p04049 (RAF1_HUMAN)	Activation of adenylate cyclase and MAPK activity, Apoptosis, cell differentiation, death-inducing signaling complex assembly
CDKN1B	Cyclin-dependent kinase inhibitor 1B	p46527 (CDN1B_HUMAN)	Cell cycle
BTRC	F-box/WD repeat-containing protein 1A	Q9Y297 (FBW1A_HUMAN)	Biological rhythms, Host-virus interaction, Ubl conjugation pathway, Wnt pathway

### Effects of hub gene expression on hepatocellular cancer prognosis

**Fig. 3:**
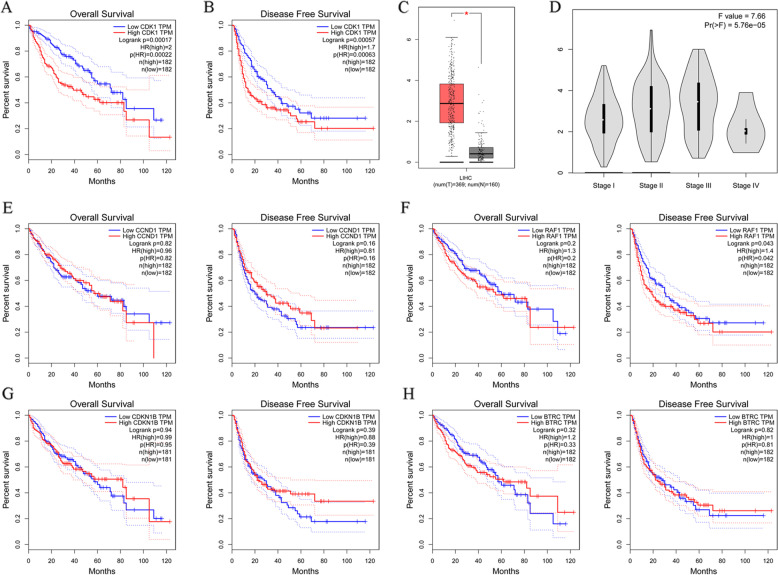
Effects of hub gene expression on prognosis of patients with hepatocellular cancer. Expression of the top 5 hub genes was assessed: CDK1, CCND1, RAF1, CDKN1B and BTRC. **A** Overall survival (OS) of patients showing high and low expression of CDK1. **B** Disease-free survival (DFS)of patients showing high and low expression of CDK1. **C** Expression of CDK1 in patients with hepatocellular cancer and healthy volunteers, *p < 0.001. **D** Expression of CDK1 in patients with hepatocellular cancer at different stages. **E** OS and DFS in patients showing high or low expression of CCND1. **F** OS and DFS in patients showing high or low expression of RAF1. **G** OS and DFS in patients showing high or low expression of CDKN1B. **H** OS and DFS in patients showing high or low expression of BTRC

We investigated the potential effects of the expression of the top 5 hub genes (CDK1, CCND1, RAF1, CDKN1B, BTRC) on the prognosis of patients with HCC. Overall survival (OS) and disease-free survival (DFS) were lower among patients showing high expression of CDK1 than among those showing low expression of CDK1 (Fig. 3A and B). The expression of CDK1 was significantly higher in patients than in healthy volunteers (Fig. 3C), primarily in patients with stage II or III liver cancer (Fig. 3D). In contrast, OS and DFS did not differ significantly between patients expressing high or low levels of CCND1, RAF1, CDKN1B or BTRC (Fig. 3E-H).

### Low arecoline concentration enhances the viability of HepG2 cells without causing ultrastructural damage

**Fig. 4:**
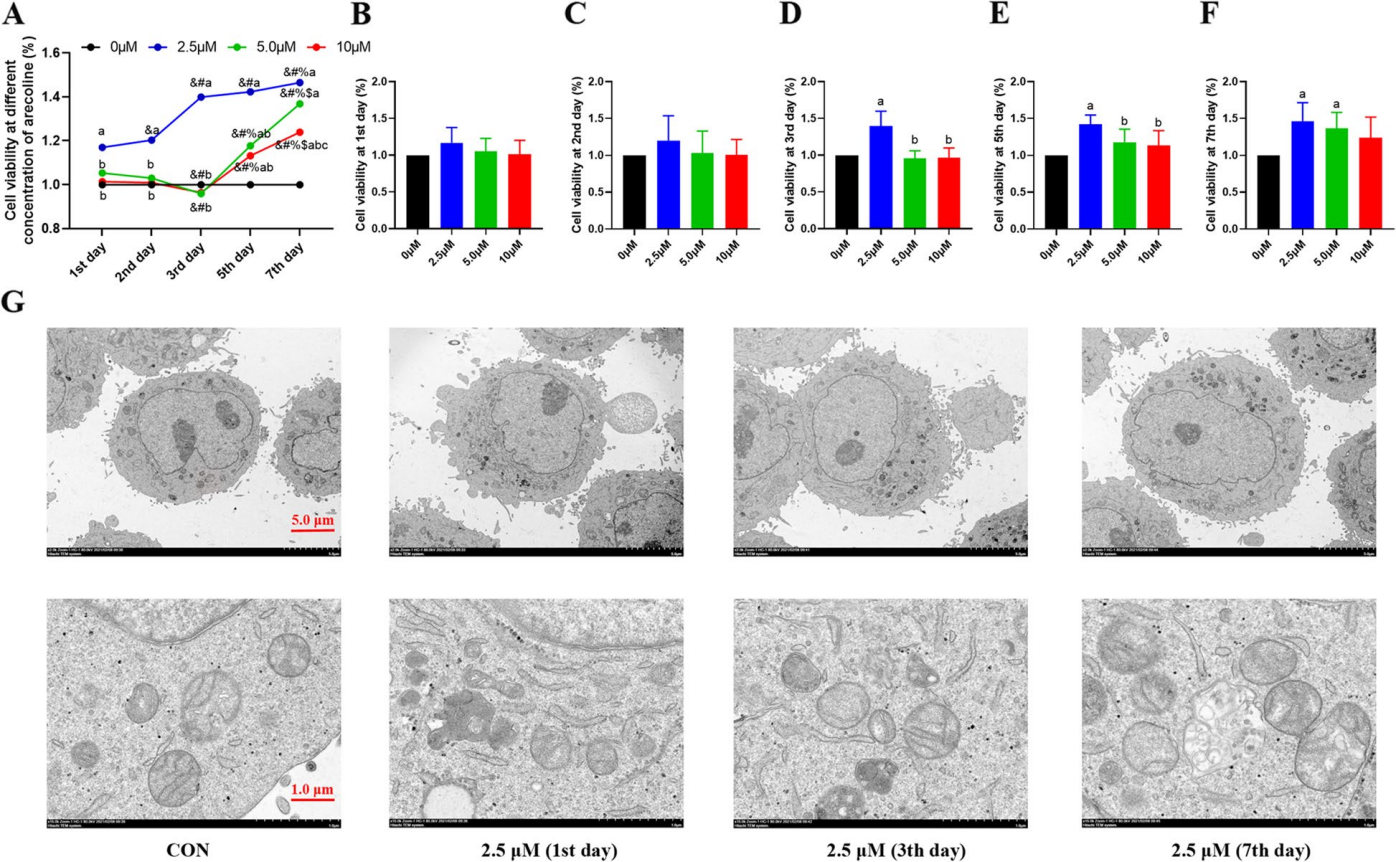
Arecoline treatment affects the cell viability and ultrastructure of HepG2 cells. **A** Changes in viability of HepG2 hepatoma cells treated with the indicated concentration of arecoline. **B-F** Quantification of the changes in HepG2 cell viability after treatment with the indicated concentration of arecoline on 1st day **B**, 2nd day **C**, 3rd day **D**, 5th day **E** and 7th day **F**. **G** Transmission electron microscopy images highlighting the ultrastructural changes in HepG2 hepatoma cells treated the indicated concentrations of arecoline. Vs 1st d, ^&^*p* < 0.05; vs 2nd d, ^#^*p* < 0.05; vs 3rd d, ^%^*p* < 0.05; vs 5th d, ^$^*p* < 0.05. Vs 0 μM treatment group, ^a^*p* < 0.05; vs 2.5 μM treatment group, ^b^*p* < 0.05

The effect of increasing concentrations of arecoline (0–10 µM) on the viability of HepG2 cells for 1–7 days was investigated using the CCK8 assay. The 2.5 µM treatment increased cell viability in a time-dependent manner, while cell viability upon 5.0 and 10 µM treatment were increased from 5 to 7th day (Fig. 4A-F). Furthermore, the 2.5 µM arecoline treatments did not induced distinctive ultrastructural cellular damage at any time point with increasing mitochondrial fusion and fission, as assessed by transmission electron microscopy (Fig. 4G).

### Low arecoline concentration promotes migration and invasion of HepG2 cells

**Fig. 5:**
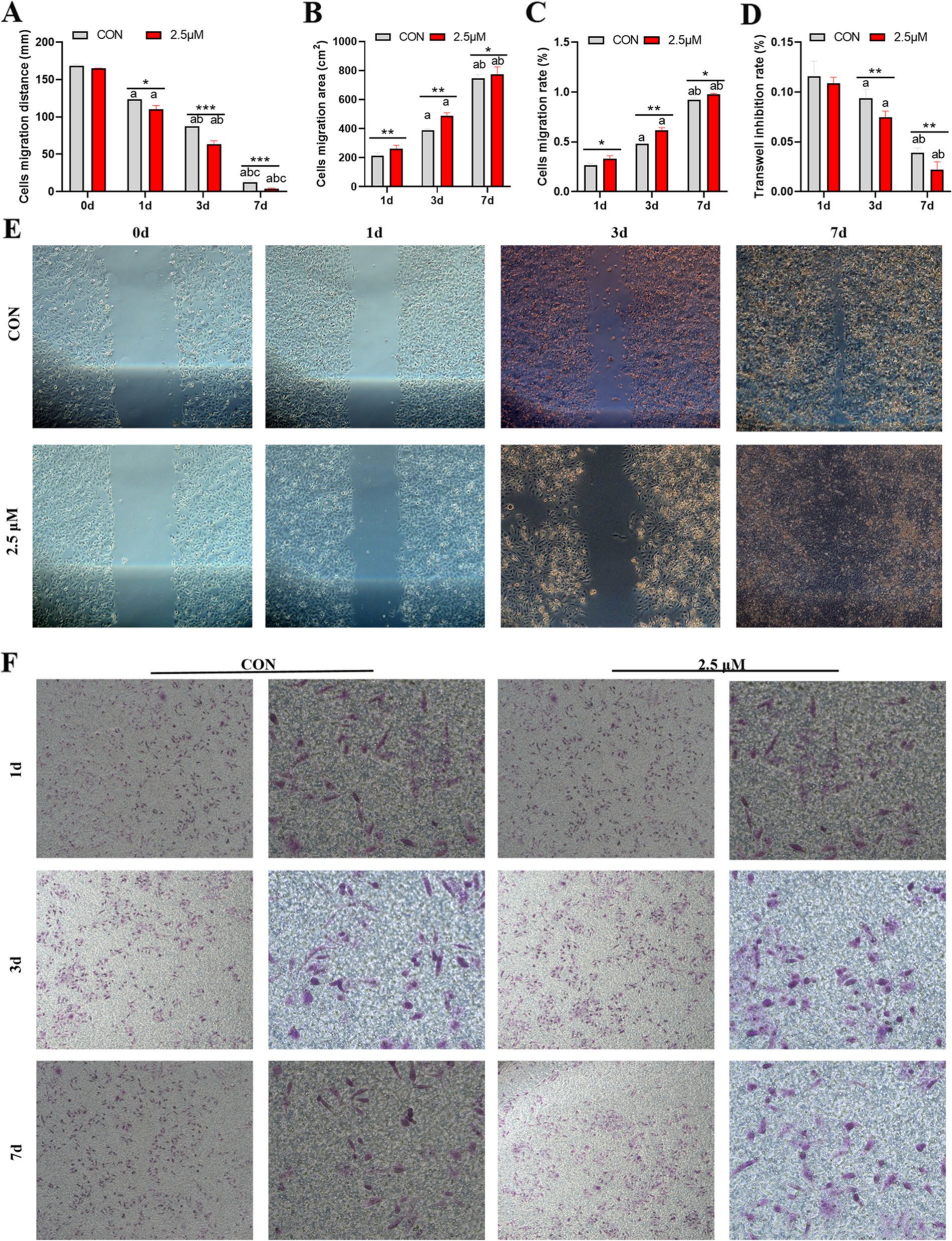
Arecoline treatment affects migration and invasion by HepG2 hepatoma cells. A Changes in scratch distance in HepG2 hepatoma cells treated with the indicated concentrations of arecoline. **B** Changes in migration area in HepG2 hepatoma cells treated with the indicated concentrations of arecoline. **C** Changes in migration rate in HepG2 hepatoma cells treated with the indicated concentrations of arecoline. **D** Changes in invasion ability in HepG2 hepatoma cells treated with the indicated concentrations of arecoline, as assessed in a transwell assay. **E–F** Migration and invasion ability of HepG2 hepatoma cells treated with the indicated concentrations of arecoline, as assessed in a wound healing Vs 0d, ^a^*p* < 0.05; vs 1d, ^b^*p* < 0.05; vs 3d, ^c^*p* < 0.05

We assessed the effects of arecoline treatment on the migration and invasion ability of HepG2 cells using wound healing and Transwell assays, respectively. Treatment of HepG2 cells with 2.5 µM of arecoline increased the migration area and rate on days 1, 3 and 7 in comparison with the 0 µM control group (Fig. 5A-C, E). The 2.5 µM treatment also increased the invasion ability in a time-dependent manner (Fig. 5D, E).

### Low arecoline concentration promotes proliferation and increases apoptosis in HepG2 cells

**Fig. 6:**
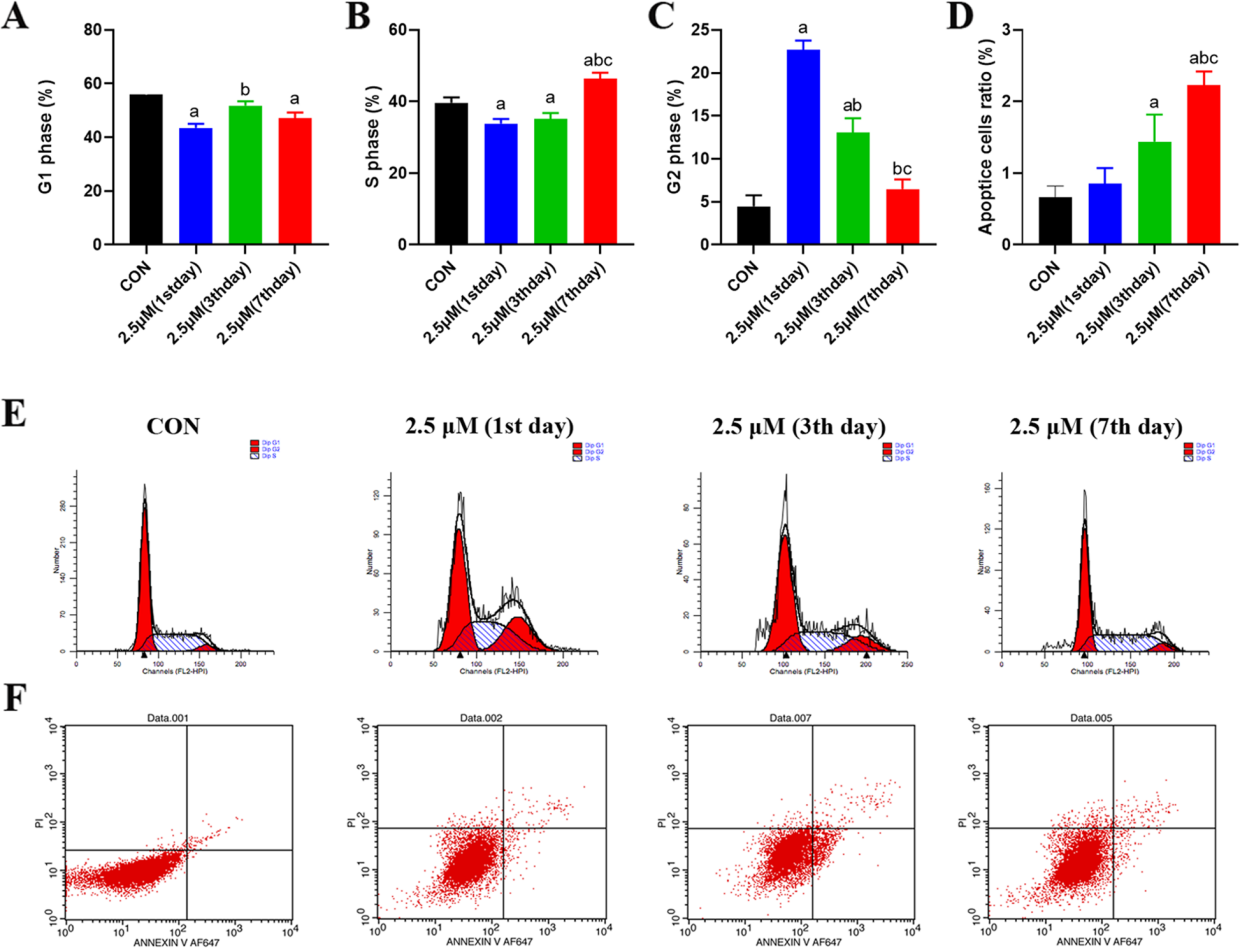
Arecoline treatment promotes cell cycle changes and affects apoptosis in HepG2 hepatoma cells. **A-C** Fraction of HepG2 hepatoma cells in the G1 **A**, S **B**, or G2 **C** phase after treatment with the indicated concentrations of arecoline. **D** Quantification of apoptosis in HepG2 cells. **E** Cell cycle in HepG2 cells on day 1, 3, 7 and con group. **F** Number of apoptotic cells after treatment with the indicated concentrations of arecoline, as assessed by annexin staining. Vs CON group, ^a^*p* < 0.05; vs 1st day, ^b^*p* < 0.05; vs3rd day, ^c^*p* < 0.05

Given the known roles of apoptosis in cancer, we investigated the effects of arecoline treatment on apoptosis in HepG2 cells using cell cycle analysis and annexin staining. We observed that treatment with 2.5 μM arecoline for 1–7 days increased the number of cells in G2 phase but decreased the number of cells in G1 and S phases (Fig. 6A-C and E). On days 1–7 after treatment with 2.5 µM arecoline, a small amount of apoptotic HepG2 cells was observed, with a time-dependent manner compared with the untreated control (Fig. 6D and F).

### Low arecoline concentration is linked to high levels of miR-21-3p, miR-21-5p and miR-1267 but is not related to IL-1β and TNF-α

**Fig. 7:**
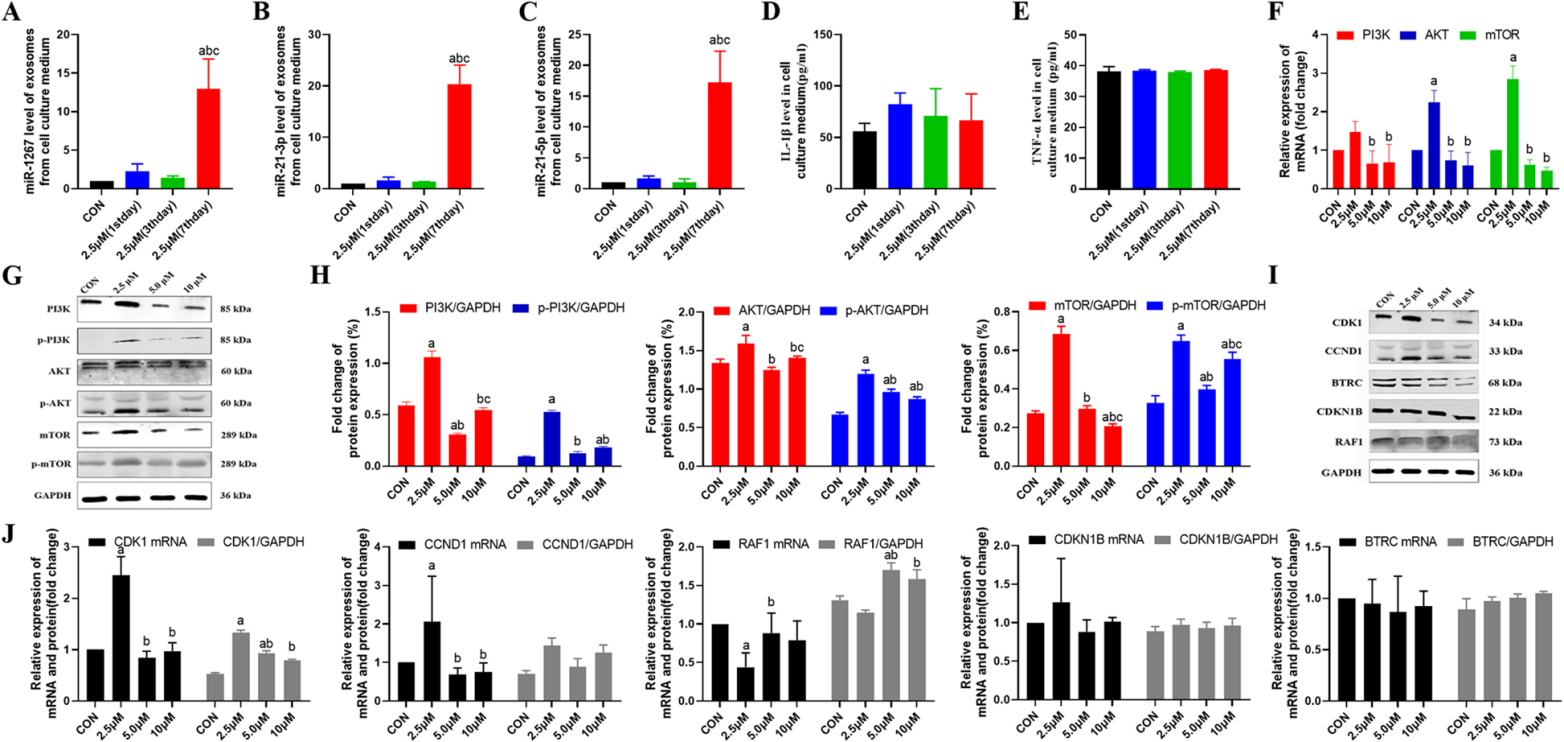
Arecoline treatment changes the expression of miRNAs and inflammatory factors via PI3K/AKT/mTOR pathwayin HepG2 hepatoma cells. All analyses were carried out with culture medium of HepG2 hepatoma cells treated with 2.5 μM arecoline and con group. **A** Exosomal miR-1267 expression. **B** Exosomal miR-21-3p expression. **C** Exosomal miR-21-5p expression. **D** IL-1β protein levels. **E** TNF-α protein levels. **F** Relative expression of mRNAs encoding PI3K/AKT/mTOR signaling proteins.**G** Western blot analysis of PI3K/AKT/mTOR signaling proteins. **H** Relative expression of PI3K/AKT/mTOR signaling proteins. **I** Western blot analysis of hub genes. **J** Relative expression of CDK1, CCND1, RAF1, CDKN1B and BTRC mRNA and protein. Vs CON group, ^a^*p* < 0.05; vs 2.5 μM group, ^b^*p* < 0.05; 5 μM group, ^c^*p* < 0.05

Exosomes are frequent carriers of miRNAs. MiR-21 and miR-1267 are the common biomarker for the tumor metastasis and recurrence, and our previous results showed that these two miRNAs are among the DE-miRNAs. After treatment with 2.5 µM arecoline for 7 days, the levels of miR-21-3p, miR-21-5p and miR-1267 in exosomes were significantly higher than in HepG2 cells treated for shorter periods. (Fig. 7A-C). With the exception of the 2.5 µM treatment, which did not lead to time-dependent IL-1β level changes (Fig. 7D). A similar trend was observed for TNF-α (Fig. 7E).

### Low arecoline concentration activates the PI3K/AKT pathway and increases the expression of CDK1 and CCND1 but downregulates RAF1 in HepG2 cells

Our results above suggested that treating HepG2 cells with 2.5 µM arecoline for 7 days significantly enhances the proliferation, migration and invasion, while inhibiting apoptosis. These results may be associated with the upregulation of miR-21-3p, miR-21-5p and miR-1267. We therefore explored the expression of the PI3K/AKT/mTOR pathway and the previously identified hub genes CDK1, CCND1, RAF1, CDKN1B and BTRC in HepG2 cells treated with 0–10 µM arecoline for 7 days.

The levels of PI3K, p-PI3K, AKT, p-AKT, mTOR, p-mTOR, CDK1 and CCND1 increased in the HepG2 cells treated with 2.5 µM arecoline for 7 days compared with control cells, and the levels were higher than in cells treated with 5.0 or 10 µM arecoline (Fig. 7F-J). Conversely, the 2.5 µM treatment reduced the expression of RAF1. Treatment with 0–10 µM arecoline did not significantly affect the expression of BTRC or CDKN1B.

## Discussion

Arecoline is the main bioactive substance in areca nuts. It can reduce atherosclerosis, regulate blood lipids and glucose, and stimulate gastrointestinal movement; and it can increase feelings of happiness, blood pressure, heart rate, saliva secretion, and improve alertness and ability to resist hunger [19]. However, the areca nut has been classified as a class 1 carcinogen and linked to oral cancer, head and neck cancer, liver cancer, breast cancer, esophageal cancer and other diseases [20–22]. Arecoline at 10–40 μM can significantly improve the migration ability of A549 and cl1-0 cells and promote the formation of F-actin cytoskeleton, a key element in cell migration [23]. In the present study, we found that 2.5 μM arecoline significantly promoted the proliferation, migration, apoptosis and invasion of HepG2 cells for up to 7 days. This may be related to the activation of the PI3K/AKT/mTOR pathway, the upregulation of CDK1 and CCND1, and the downregulation of RAF1.

Our bioinformatic results showed that miR-21-3p, miR-21-5p and miR-1267 are upregulated in arecoline related cancer cells. The expression of miR-21-3p, miR-21-5p and miR-1267 in the exosomes from culture medium was also significantly elevated in HepG2 cells after treatment with 2.5 μM arecoline for 7 days. Thus, these exosomal miRNAs may play important roles in arecoline-driven proliferation, migration and invasion of hepatoma cells. Our results extend previous work on these miRNAs. Levels of circulating miR-21 are significantly upregulated in patients with HCC, and the levels are related to the clinic opathological parameters of tumor capsule infiltration and tumor-node-metastasis classification. Moreover, patients with upregulated miR-21 levels show worse prognosis [24]. The inhibition of miR-21-3p has been proposed to sensitize hepatocellular carcinoma stem cells to trail by de-repressing the miR-21-3p/PTEN axis and PI3K/AKT/Bad cascade [25]. The miR-21-5p is highly expressed in hepatocellular carcinoma tissues and cell lines, especially in patients with cisplatin-resistant HCC [14, 26, 27]. The expression of miR-21 in serum exosomes is significantly upregulated in hepatocellular carcinoma, pointing to its potential role in hepatocellular carcinoma metastasis and recurrence [25, 27]. For its part, miR-1267 may inhibit the development of breast cancer, while promoting invasion and metastasis [28]. We are unaware that miR-1267 has been linked to metastasis, recurrence or prognosis in HCC.

In vitro studies, we found that treating HepG2 cells with 2.5 μM arecoline enhanced cell viability at day 3 and 7, increased G1 and S phase but decreased G2 phase, and upregulated CDK1 and CCND1. CDK1 and CCND1 are the key enzymatic complex required for mitosis, and normally activated to regulate the cell cycle of hepatocellular carcinoma cell adhesion [29–31]. It has been reported that CDK1 is a novel mediator of apoptosis resistance in BRAFV600E colorectal cancers whose dual targeting with a MEK inhibitor may be therapeutically effective [32]. These results suggested that HepG2 cells favors high cell viability and active mitosis after treatment with 2.5 μM arecoline, hence we think 2.5 uM arecoline promotes proliferation and apoptosis of HepG2 cells.

We also proved that treating HepG2 cells with 2.5 μM arecoline upregulated all three key players of the PI3K/AKT/mTOR pathway. The PI3K/AKT/mTOR pathway plays crucial roles in the proliferation, differentiation and invasion of hepatoma cells. The activation of this pathway in HepG2 cells is related to lipid metabolism, which also strongly influences the proliferation, migration and invasion of hepatoma cells and is regulated by PCSK9 and SREBP2 [33]. Inhibition of the mTOR/PI3K pathway and induction of caspase-dependent apoptosis can improve liver function, oxidative DNA damage and tumor-specific markers, thus counteracting the carcinogenic effect of diethylnitrosamine [34]. Knocking out the CaMKKβ gene in HepG2 cells inhibits the proliferation, invasion and glycolysis of HepG2 cells while increasing apoptosis, and these effects involve inhibition of the PI3K/AKT pathway [35]. Treating rat hepatoma cells with arecoline activates ATM/atr-p53-p21 (WAF1) and PI3K/AKT-mtor-p53 pathways were significantly activated [9, 36]. Our results support and extend previous studies implicating the PI3K /AKT/mTOR pathway in arecoline-induced oncogenesis.

We also found that the expression of CDK1 and CCND1 was upregulated in HepG2 hepatoma cells following a 2.5 μM arecoline treatment. Overexpression of CDK1 and CCND1 is linked with a low survival rate in HCC [37]. Metformin can arrest cells in G2/M phase by reducing the expression of CDK1 and ultimately inhibiting the proliferation of hepatoma cells [38]. It was also found in our experiment that 2.5 μM arecoline promoted most of HepG2 cells to stay in G2/M phase. These observations implicate CDK1 in the cellular proliferation process in hepatoma. MiR-582-5p regulates the metastasis and recurrence of HCC by directly inhibiting the expression of CDK1 and AKT3 and indirectly inhibiting the expression of CCND1 [39]. CCND1 inhibition, regulated by miR-144, can significantly inhibit the proliferation, migration and invasion of hepatoma cells [40]. CCND1/CDK1-mediated phosphorylation drives the G2/M conversion and shortens the whole cell cycle time [41]. These results indicate that CDK1 and CCND1 play a key role in the cell progression of hepatocellular carcinoma, and their high expression is closely linked with the promotion of proliferation, migration and invasion of such tumor cells.

We found that the expression of RAF1 was downregulated in HepG2 cells treated with 2.5 μM arecoline. The RAF1 gene encodes a MAP kinase, which acts downstream of the Ras family of membrane-associated GTPases. Once activated, RAF1 can be phosphorylated to activate the specific protein kinases MEK1 and MEK2, which can in turn activate the serine/threonine specific protein kinases ERK1 and ERK2. Activated ERK is a pleiotropic effector of cell physiology, and it plays an important role in controlling gene expression involved in cell division, apoptosis, cell differentiation and cell migration [42]. RAF1 is a negative regulator of hepatocarcinogenesis; downregulating RAF1 promotes the proliferation of human hepatocellular carcinoma in xenografts and cell culture, and it promotes the proliferation of mouse hepatoma cells and hepatocarcinogenesis [43]. Expression of RAF1 is lower in HCC than in peripheral non-tumor tissues, and the magnitude of this difference negatively correlates with tumor grade [43]. Silencing RAF1 by shRNA can promote the proliferation of Hep3B cells [44]. Knocking down RAF1 with doxycycline leads to enlargement of mouse hepatocellular carcinoma xenograft [45]. Our results support and extend previous studies that indicate that RAF1 downregulation promotes the proliferation and migration of hepatocellular carcinoma cells.

Nevertheless, our results should be interpreted with caution given the limitations of our study. First, we examined only cells in culture, so further studies should verify our findings in vitro. Second, we did not confirm the role of PI3K/AKT/mTOR pathway or the roles of CDK1, CCND1 and RAF1 in gene knockdown experiment. We plan to explore how PI3K, AKT, CDK1, CCND1 and RAF1 contribute to arecoline-induced liver cancer using a rat intrahepatic tumor transplantation model and siRNA interference. Third, we just inferred that miR-21-3p, miR-21-5p and miR-1267 in the exosomes may be the mechanisms of arecoline promoting the proliferation, migration and invasion of hepatoma cells.

Despite these limitations, a low dose of arecoline (2.5 μM) can promote the proliferation, migration and invasion of HepG2 hepatoma cells. We found this to be linked to the increased levels of miR-21 and mir-1267 in exosomes, activation of the PI3K/AKT/mTOR pathway, upregulation of CDK1 and CCND1, and downregulation of RAF1.

## Conclusion

In summary, CDK1, CCND1, RAF1, CDKN1B and BTRC were defined as top 5 hub target-genes, and the patients with hepatocellular cancer with high expression of CDK1 showed poor prognosis. 2.5 μM arecoline can improve cell viability of HepG2 cell, promote cell proliferation, migration and apoptosis to a certain extent. miR-21 and miR-1267 might play an essential role in arecoline-promoted PI3K/AKT/mTOR activation and migration in HepG2 cells with upregulation of CDK1, CCND1 and downregulation of RAF1.

## Data Availability

All data and materials generated or analyzed during this study are included in the article.

## Abbreviations

DE-miRNAs: Deferentially expressed miRNAs
HCC: Hepatocellular cancer
CYP2E1: Cytochrome p450 2E1
miRNAs: MicroRNAs
GEO: Gene Expression Omnibus
KEGG: Kyoto Encyclopedia of Genes and Genomes
BPs: Biological processes
CCs: Cellular components
MFs: Molecular functions
CCK: Cell Counting Kit
IL: Interleukin
TNF: Tumor necrosis factor
PBS: Phosphate buffered saline
RT-qPCR: Real time-quantitative polymerase chain reaction
